# Effect of liraglutide biosimilar vs. reference liraglutide on weight reduction in T2DM patients with obesity: post hoc analysis of phase III trial

**DOI:** 10.1186/s40842-025-00219-7

**Published:** 2025-03-25

**Authors:** Sujoy Ghosh, Bipin Sethi, Sanjay Kalra, Manash P. Baruah, Abhishek Mane, Sanjay Choudhari, Anup Petare, Mayur Jadhav, Saiprasad Patil, Hanmant Barkate

**Affiliations:** 1 Manipal Hospitals, Kolkata, India; 2 Care Hospitals, Hyderabad, India; 3 Bharti Research Institute of Diabetes & Endocrinology, Karnal, India; 4 Apollo Hospitals, Guwahati, India; 5 Glenmark Pharmaceuticals Ltd, Mumbai, India

**Keywords:** Liraglutide biosimilar, Reference liraglutide, Obesity, BMI, Weight reduction

## Abstract

**Background:**

Obesity is a chronic metabolic disease of global concern, often associated with Type 2 Diabetes Mellitus (T2DM). Global guidelines recommend holistic approach for T2DM management by addressing the associated comorbidities. Here, we have conducted a post-hoc evaluation of Liraglutide biosimilar Phase III trial on weight reduction and glycaemic benefits in Indian T2DM patients with obesity in comparison to reference liraglutide.

**Methods:**

We have conducted a post-hoc analysis of Liraglutide biosimilar Phase III trial on weight reduction in Indian T2DM patients with obesity in comparison to reference liraglutide. We evaluated weight reduction and HbA1c improvement in Indian T2DM patients (BMI > 25 kg/m^2^) from baseline to week 24. Group A – Intervention arm: Liraglutide Biosimilar in T2DM patients with obesity Group B – Control arm: Reference Liraglutide in T2DM patients with obesity. Primary endpoint was mean change in body weight from baseline to week 24.

**Results:**

179 T2DM patients (BMI > 25 Kg/m^2^ and above) who satisfied the inclusion criteria, were included in this post-hoc analysis. The mean BMI of T2DM patients with obesity in Biosimilar Liraglutide arm was 29.8 ± 4.6 kg/m^2^ and that in the Reference Liraglutide arm it was 29.8 ± 4.8 kg/m^2^. Significant mean weight reduction (Mean ± SD) of 5.5 ± 1.2 kg (7.3 ± 1.7%) and 7.1 ± 2.6 kg (8.9 ± 1.7%) (*p* < 0.001) was demonstrated by both biosimilar liraglutide and reference liraglutide respectively. However, weight reduction was comparable across both the groups at week 24 (*p* = 0.71). Likewise, glycaemic parameters (HbA1c, FPG and PPG) significantly improved in both the treatment arms (*p* < 0.001). However, they were comparable across the groups at week 24 with a p value of 0.89, 0.43 and 0.17 for HbA1c, FPG and PPG respectively.

**Conclusion:**

Biosimilar Liraglutide at a dose of up to 1.8 mg was non-inferior to reference Liraglutide and resulted in significant weight reduction and glycemic control (HbA1c, FPG and PPG) in Indian T2DM patients with obesity.

**Supplementary Information:**

The online version contains supplementary material available at 10.1186/s40842-025-00219-7.

## Introduction

Obesity is chronic metabolic disease that has emerged as a global public health problem [1]. Type 2 Diabetes Mellitus (T2DM) in association with obesity, usually known as “Diabesity” is an alarming combination that significantly increases both morbidity as well as mortality [2]. Patients’ health-related quality of life is further compromised by various obesity-related comorbidities including dyslipidaemia, hypertension, Metabolic dysfunction Associated Fatty Liver Disease (MAFLD), Obstructive Sleep Apnoea (OSA), Cardiovascular Disease (CVD), Cancer, Osteoarthritis and others [3] More than 88% of People living with Diabetes (PwD) are suffering from overweight or obesity [4]. Recently published data from the ICMR-INDIAB 17 study stated the prevalence of generalized obesity (BMI > 25 kg/m^2^) and that of abdominal obesity (waist circumference > 90 cm for men and > 80 cm for women) at 28.6% and 39.5% respectively [5]. 

Holistic management for diabetes includes lifestyle modification and dietary changes combined with physical activity and behavioural interventions. These recommendations are advocated by American Diabetes Association (ADA), European Association for Study of Diabetes (EASD), American Association of Clinical Endocrinologists (AACE) for management of T2DM patients with obesity [6, 7]. Patient centric approach and holistic management of T2DM with obesity can help improve patient outcomes in the presence of “Diabesity” [8]. Weight loss of 5 to 10% has been shown to reduce complications related to obesity and improve quality of life along with significant health benefits [9–11].

By virtue of their extra glycaemic benefits, specifically by promoting weight reduction and satiety control, glucagon-like peptide-1 receptor agonist (GLP-1RA) are recommended as the 1st line therapy by most international organizations, for weight management in T2DM patients [7]. Liraglutide, a GLP-1RA, is approved for chronic weight management (at a dose of 3.0 mg) in individuals with BMI ≥ 27 kg/m^2^ with one weight-related comorbidity or in people with obesity (BMI ≥ 30 kg/m^2^) [12, 13]. The SCALE clinical trial [13] documented weight loss benefits of Liraglutide with 3 mg dose in patients with obesity with or without diabetes However, the same trial also documented a significant weight loss of 5 kg with 1.8 mg liraglutide. Likewise, literature has demonstrated the beneficial effects of low dose Liraglutide (1.8 mg) on weight reduction [14, 15]. Recently, Liraglutide Biosimilar has received marketing authorization for management of T2DM in India at a maximum dose of up to 1.8 mg. Phase III trial evaluated the glycemic efficacy of the Liraglutide Biosimilar in comparison to the reference Liraglutide on background Oral Antidiabetic Drug (OAD) therapy [16]. However, to evaluate its effect in T2DM patients with obesity we conducted this post hoc analysis of Phase III trial.

## Materials and methods

### Study design

The study design of Phase III clinical trial of Liraglutide biosimilar in comparison with reference liraglutide, their detailed methods along with the primary results have been published previously [16] wherein 17 clinical trial sites throughout India participated in this randomized, open label, parallel group, two-arm study. Assessors were blinded and the efficacy, safety, and tolerability of liraglutide Biosimilar developed by Levim Biotech LLP, was compared to the reference liraglutide in the treatment of T2DM patients already on a background stable therapy of other oral hypoglycaemic agents (CTRI/ 2022/02/040261).

**Fig. 1:**
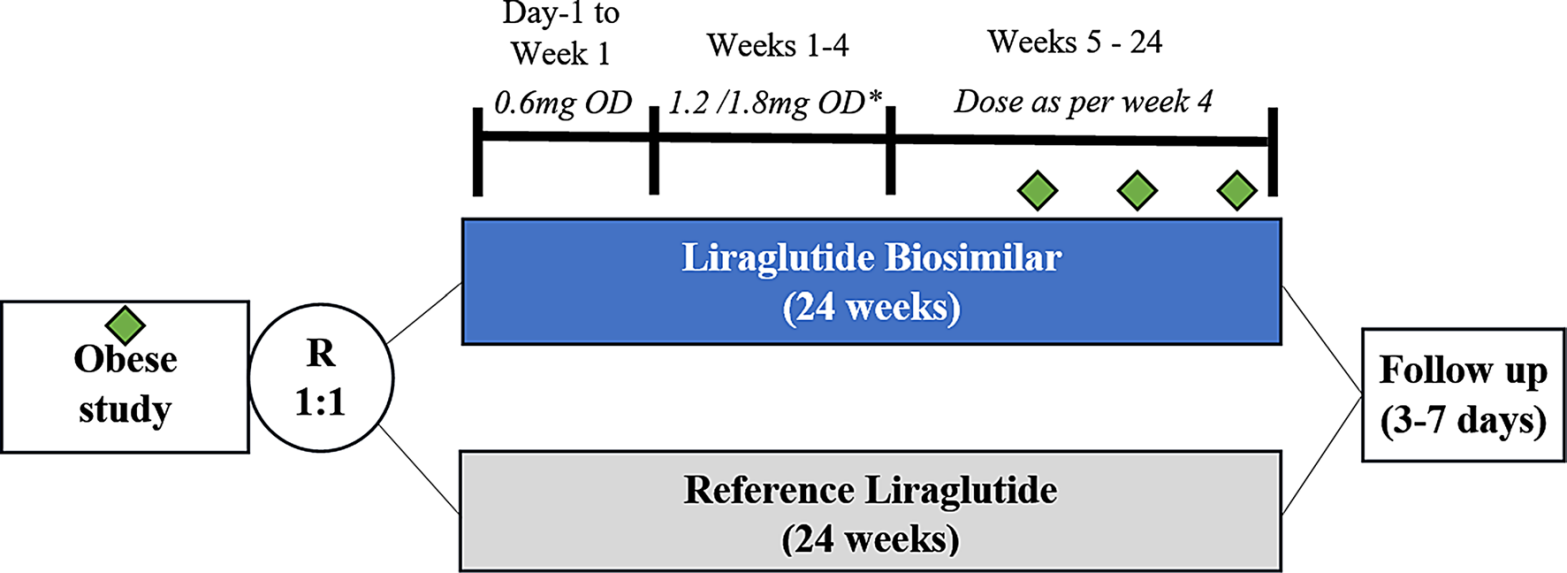
Liraglutide biosimilar Phase III Study flow diagram. HbA1c% (rhombus) was measured at baseline (screening), week 12, week 18 and week 24. Source: Supplementary material, Phase III trial of Liraglutide Biosimilar, Diabetes Research and Clinical Practice, Volume 207, 111,034

The study population consisted of T2DM patients of either sex, from 18 to 65 years of age with baseline HbA1c levels between 7 and 10%. A total of 256 T2DM patients were randomized in 1:1 ratio to receive either liraglutide biosimilar (1.2 mg/day or up titrated to the maximum dose of 1.8 mg/day) or reference liraglutide (1.2 mg/day or up titrated to the maximum dose of 1.8 mg/day), both in addition to standard-of-care therapy. The treatment duration was of 24 weeks excluding the screening period. Flow diagram depicting the study design is given in Fig. 1.

All patients enrolled were initiated on either Biosimilar or Reference Liraglutide at a starting dose of 0.6 mg initially. Dose titration was done after minimum of 1 week of treatment with 0.6 mg dose for both treatment arms. Following the 1st dose titration to 1.2 mg, the dose was up titrated by the investigators to 1.8 mg to achieve better glycaemic control if targets were not met, subject to tolerance/side effects. Dose escalation, (to 1.8 mg dose) was done after at least 1 week of treatment with 1.2 mg dose based on the 6-point self-monitoring of blood glucose (SMBG) profile values. Patients who tolerated 1.2 mg dose and who were being considered for titrating the dose to 1.8 mg were instructed to do SMBG after each meal i.e. breakfast, lunch and dinner. The clinical study protocol, informed consent forms, and all other study-related documents were approved by independent ethics committees or institutional review boards, as applicable. The study was conducted in compliance with the protocol, the ethical principles were followed in accordance with the Declaration of Helsinki, International Council for Harmonization (ICH) consolidated Guideline the New Drugs and Clinical Trial Rules, 2019 (CDSCO, India) and other applicable regulatory requirements.

### Subgroup for post hoc analysis

**Fig. 2:**
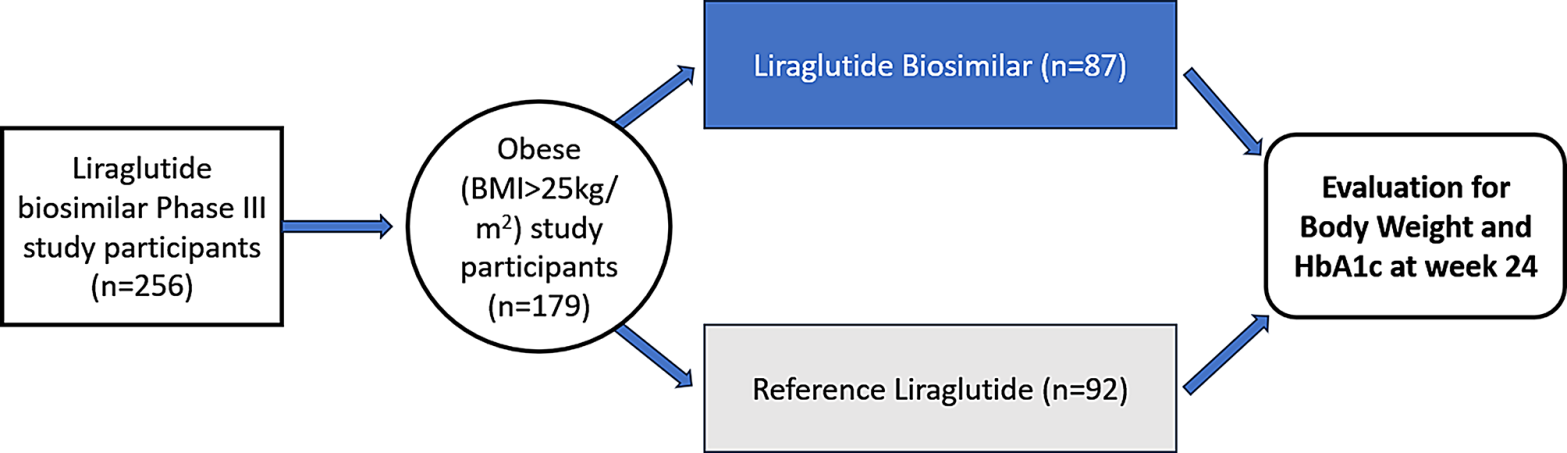
Post hoc analysis study flow diagram

Post hoc analysis of the Liraglutide biosimilar phase III trial was done to evaluate its effect on weight and HbA1c reduction across both the treatment arms from baseline to week 24 in T2DM patients with obesity. Study participants with BMI > 25 kg/m^2^ were classified as to be suffering from obesity (Asia Pacific guidelines for obesity) [17] These individuals were divided into two groups as follows: Group A – Intervention arm: Liraglutide Biosimilar in T2DM patients with obesity and Group B – Control arm: Reference Liraglutide in T2DM patients with obesity. The subgroups included for analysis in our study are depicted in Fig. 2.

### Study endpoints

The primary efficacy endpoint for this post hoc analysis was mean change in body weight from baseline to week 24 across both the treatment arms. The secondary endpoints included mean changes from baseline to week 24 in HbA1c, fasting plasma glucose (FPG) and post-prandial glucose (PPG).

### Statistical analysis

Raw data of the study participants was extracted and tabulated as per BMI stratification. We used SPSS software version 23, San Diego CA, for data analysis. Demographic characteristics of all the patients were captured and baseline comparison was done. Continuous variables were represented as Mean ± SD. Post normality assessment, within group analysis was done using ‘paired t’ test while intergroup comparison was done using independent sample t test. A p value of < 0.05 was statistically significant.

## Results

### Demographic characteristics

179 T2DM patients with obesity (BMI 25 Kg/m^2^ and above) who satisfied the inclusion criteria, were included amongst the 256 patients from phase III clinical trial for Liraglutide Biosimilar. 87 patients received biosimilar liraglutide while 92 patients were on reference liraglutide. The mean BMI of T2DM patients with obesity in the Biosimilar Liraglutide arm was 29.8 ± 4.6 kg/m^2^ and in the Reference Liraglutide, arm was 29.8 ± 4.8 kg/m^2^. A total of 97 patients were on 1.2 mg dose (53 on biosimilar liraglutide and 44 on reference liraglutide), while the remaining 82 patients required dose up titration to 1.8 mg (34 on biosimilar liraglutide and 48 on reference liraglutide). Baseline demographic characteristics of patients have been mentioned in Table 1.

**Table 1:** Demographic characteristics of the study participants as per the subgroups

Sr. no	Parameter	Treatment		T2DM patient with obesity (Mean ± SD) / *n*	*p* value
1	Age	Biosimilar Liraglutide		50.9 ± 8.0	0.85
		Reference Liraglutide		51.1 ± 7.5	
2	Gender composition	Biosimilar Liraglutide	Male	30	NA
			Female	57	
		Reference Liraglutide	Male	50	NA
			Female	42	
3	Height	Biosimilar Liraglutide		160.1 ± 7.3	0.24
		Reference Liraglutide		162.7 ± 8.4	
4	Weight	Biosimilar Liraglutide		76.4 ± 12.3	0.19
		Reference Liraglutide		79.1 ± 15.2	
5	BMI	Biosimilar Liraglutide		29.8 ± 4.6	0.93
		Reference Liraglutide		29.8 ± 4.8	

### Weight parameters

**Fig. 3:**
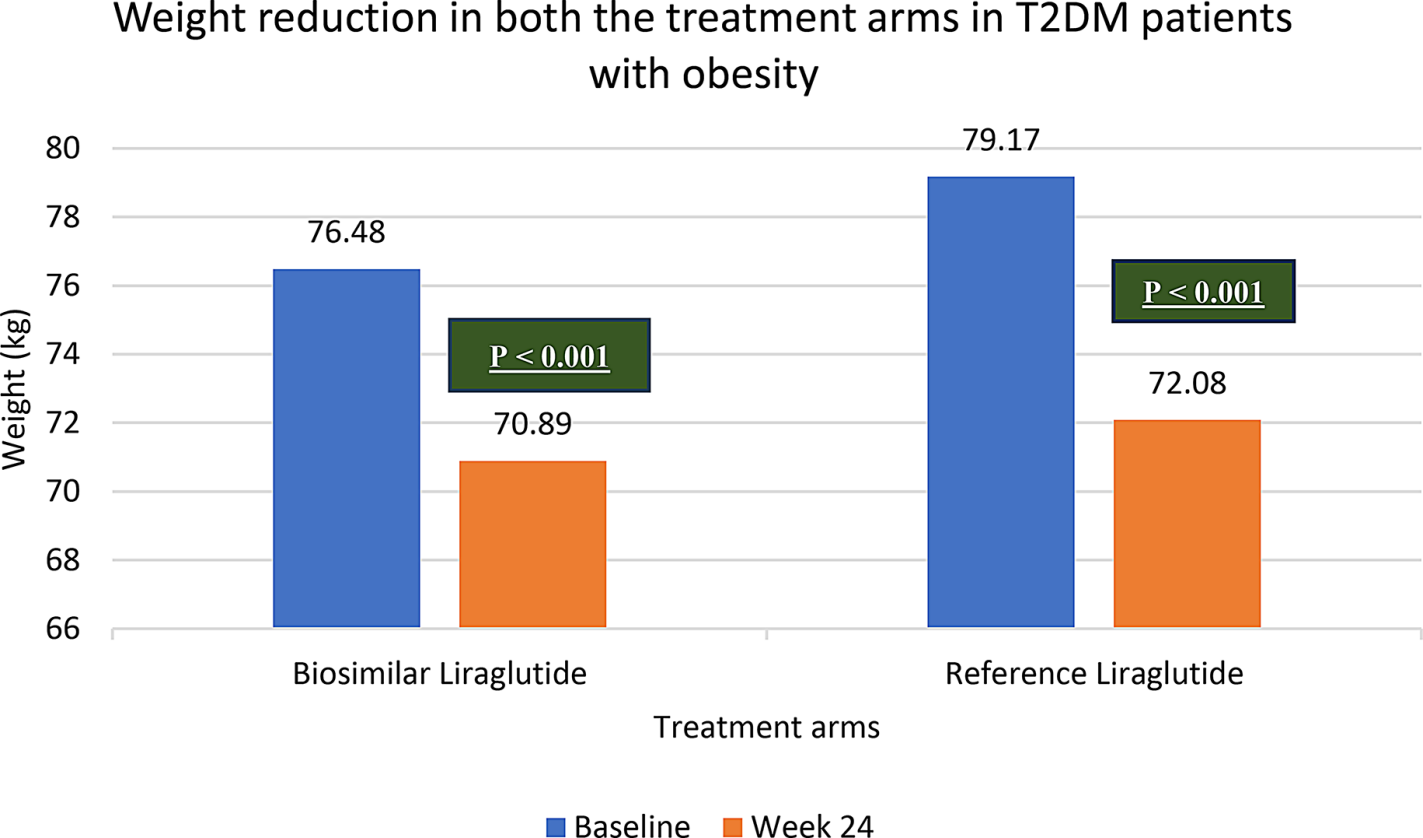
Change in weight in T2DM patients with obesity (BMI > 25 kg/m^2^) (Intra group)

The mean weight of patients in both the treatment arms at baseline and week 24 has been given in Table 2. Average weight reduction of 5.5 ± 1.2 kg (7.3 ± 1.7%) and 7.1 ± 2.6 kg (8.9 ± 1.7%) from baseline to week 24, was demonstrated by biosimilar liraglutide and reference liraglutide respectively (Mean ± SD). Treatment with biosimilar liraglutide was associated with a weight loss of at least 5%, 10% and 15% in 21%, 8% and 2% of the patients respectively. Likewise, with reference liraglutide, 17% and 2% patients lost at least 5% and 10% body weight respectively, while none of the patient could achieve a weight loss of over 15%. Both biosimilar and reference liraglutide demonstrated significant weight reduction from baseline to end of study. (*p* < 0.001). However, weight reduction between both the groups at week 24 with a *p* = 0.71 was comparable. The results are demonstrated in Fig. 3 and tabulated in Table 3.

**Table 2:** Change in weight in T2DM patients with obesity (BMI > 25 kg/m^2^) (Intra group)

Parameter	Treatment	Baseline (Mean ± SD)	Week 24 (Mean ± SD)	Change from baseline to week 24 (Mean ± SD)	Percentage change from baseline (Mean ± SD)	*p* value
Weight	Biosimilar Liraglutide	76.4 ± 12.3	70.8 ± 19.5	5.5 ± 1.2	7.3 ± 1.7	< 0.001
	Reference Liraglutide	79.1 ± 15.2	72.1 ± 23.6	7.1 ± 2.6	8.9 ± 1.7	< 0.001

**Table 3:** Weight reduction in T2DM patients with obesity (BMI > 25 kg/m^2^) (Inter group)

Parameter	Treatment	Week 24 (Mean ± SD)	*p* value
Weight	Liraglutide Biosimilar	7.3 ± 1.7	**0.71**
	Reference Liraglutide	8.9 ± 1.7	

### Glycemic parameters

**Fig. 4:**
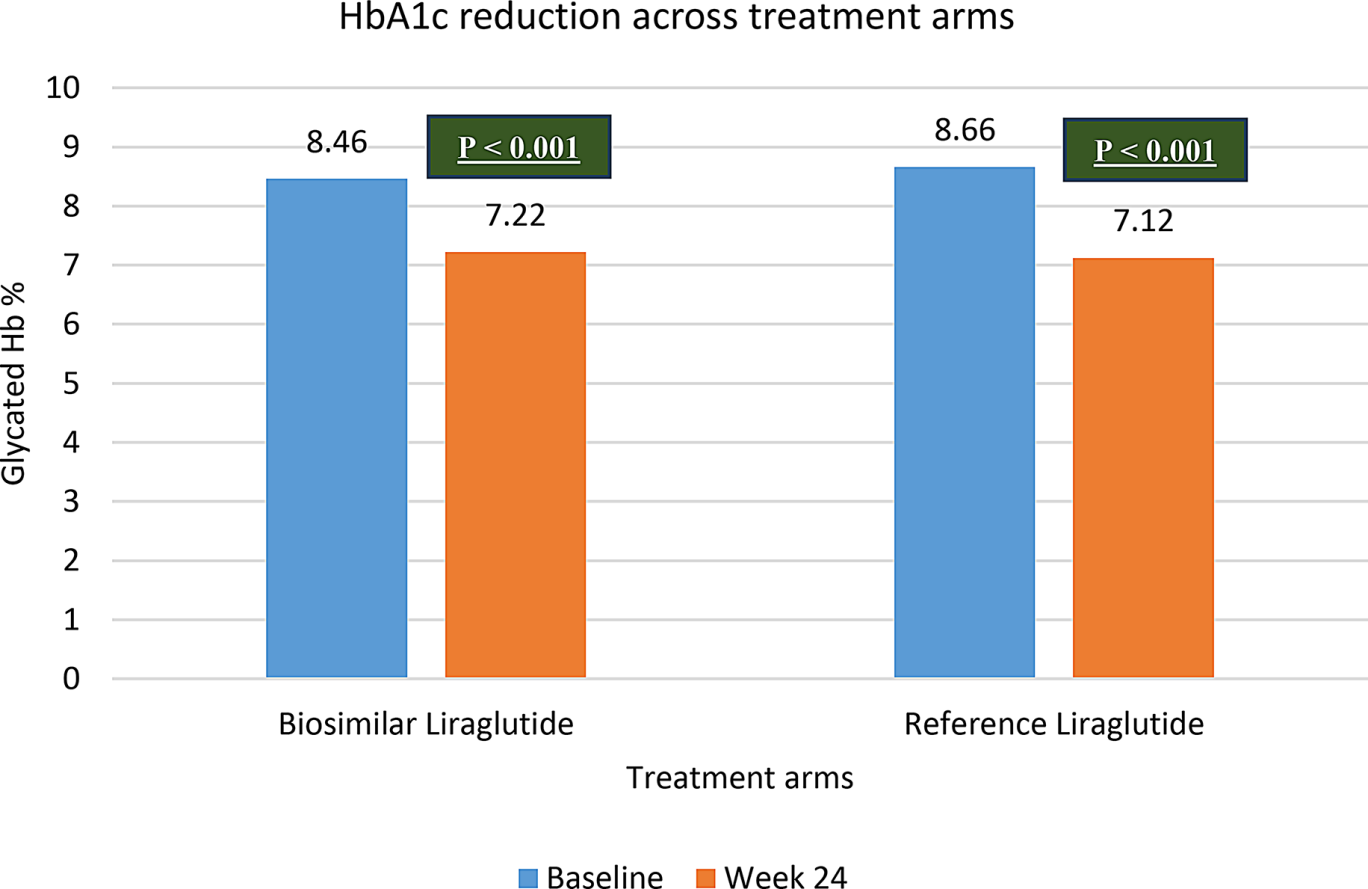
HbA1c changes across T2DM patients with obesity (BMI > 25 kg/m^2^)

**Fig. 5:**
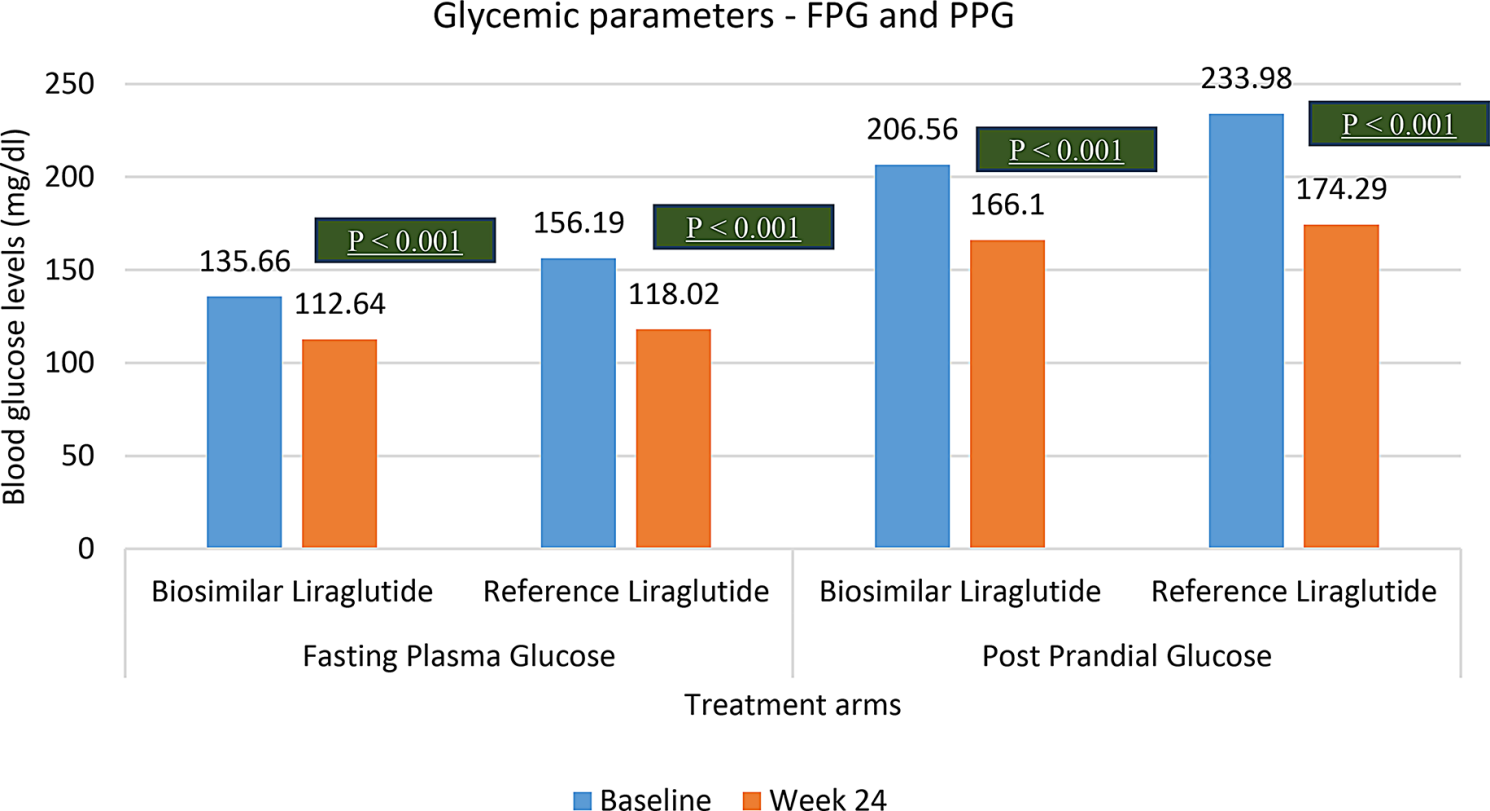
FPG and PPG changes across T2DM patients with obesity (BMI > 25 kg/m^2^)

Glycemic parameters like HbA1c, FPG and PPG demonstrated a significant change from baseline to week 24 across both Liraglutide biosimilar and Reference liraglutide study arms. (*p* < 0.001). The improvement in glycemic parameters have been represented in Fig. 4 and depicted in Table 4. However, HbA1c, FPG and PPG across Liraglutide biosimilar and Reference liraglutide at week 24 were comparable. The p value for HbA1c, FPG and PPG was 0.89, 0.43 and 0.17 respectively (Table 5; Fig. 5).

**Table 4:** Glycemic parameters in T2DM patients with obesity (Intra group)

Sr. no	Parameter	Treatment	Baseline (Mean ± SD)	Week 24 (Mean ± SD)	*p* value
1	HbA1c	Biosimilar Liraglutide	8.4 ± 0.8	7.2 ± 1.9	< 0.001
		Reference Liraglutide	8.6 ± 0.8	7.1 ± 2.1	< 0.001
2	FPG	Biosimilar Liraglutide	135.6 ± 38.7	112.6 ± 35.2	< 0.001
		Reference Liraglutide	156.1 ± 45.5	118.1 ± 44.1	< 0.001
3	PPBG	Biosimilar Liraglutide	206.5 ± 53.4	166.1 ± 51.9	< 0.001
		Reference Liraglutide	233.9 ± 63.7	174.2 ± 61.4	< 0.001

**Table 5:** Glycemic parameters across T2DM patients with obesity (Inter group)

Sr. no	Parameter	Treatment	Week 24 (Mean ± SD)	*p* value
1	HbA1c	Biosimilar Liraglutide	7.22 ± 1.92	**0.898**
		Reference Liraglutide	7.12 ± 2.14	
2	FPG	Biosimilar Liraglutide	112.64 ± 35.28	**0.433**
		Reference Liraglutide	118.02 ± 44.19	
3	PPBG	Biosimilar Liraglutide	166.10 ± 51.94	**0.178**
		Reference Liraglutide	174.29 ± 61.40	

## Discussion

Obesity and T2DM are associated with adverse clinical outcomes. Major guidelines throughout the globe have recommended GLP-1RA for management of Obesity alone, as well as for T2DM with Obesity. Globally, Liraglutide has been extensively studied for its extra glycaemic benefits mainly pertaining to weight loss. The primary objective of our post hoc analysis was to evaluate the efficacy of Levim Biotech LLP developed Biosimilar liraglutide compared to the Reference liraglutide in weight reduction over 24 weeks’ duration in T2DM patients along with obesity. Based on post hoc results that we have analysed, Biosimilar liraglutide was found to be non-inferior to the Reference liraglutide with respect to change in mean weight and HbA1c values from baseline to end of the study.

Our results with lower dose of Liraglutide Biosimilar (1.8 mg) significantly reduced weight from baseline to end of study. SCALE randomized clinical trial program evaluated the efficacy of different doses of Liraglutide on weight reduction in patients with obesity [13]. Liraglutide low dose (1.8 mg) and high dose (3 mg) was compared with placebo, in terms of absolute weight reduction. Patients on 3 mg Liraglutide demonstrated 6.4 kg weight loss, while those on 1.8 mg lost 5 kg weight. The weight loss with 1.8 mg with Biosimilar Liraglutide in T2DM patients with obesity in our study was 5.5 kg. Weight reduction in terms of percentage was 4.7% observed with 1.8 mg liraglutide dose in SCALE clinical trial. Our study demonstrated 7.9% weight reduction with 1.8 mg dose. The findings for weight reduction were consistent with the findings from the SCALE clinical trial using 1.8 mg liraglutide.

Globally, 3 mg Liraglutide has been approved for management of Obesity in addition to dietary modification and lifestyle interventions. It has been observed that higher the dose of GLP-1RA, greater is its weight reducing efficacy. Weight reduction of around 8.3% has been observed with Liraglutide 3 mg dose in the western population. Our study demonstrated a weight loss of 7.9% with 1.8 mg dose, comparable with the degree of weight reduction achieved with higher dose of liraglutide [13]. Higher GLP-1RA dose is particularly effective in western population who have higher baseline BMI with higher body fat content [18]. 

Studies have shown that use of higher dose of GLP-1RA might lead to lean muscle mass wasting to certain extent in comparison to the amount of fat tissue being lost [19]. Especially in Asian Indian T2DM patients who typically have a phenotype characterized by lean body composition with abdominal obesity [20]. In such phenotype, even lower dose of Liraglutide (1.8 mg) is associated with considerable weight reduction [12, 17]. Moreover, higher prevalence of overweight and prediabetes in Asian Indians point to an ongoing acceleration of both obesity and T2DM. It is of particular interest that Asian Indians are more likely than white Caucasians to develop obesity and diabetes at younger age with quick disease progression. Keeping this perspective in mind and all the non-communicable diseases (NCD) that come along with T2DM patients with obesity, body weight reduction along with adequate glycaemic control offers a promising approach for holistic management of T2DM patients with obesity [21, 22]. 

In addition to all the factors discussed above, expensive cost of GLP-1RA medications was an important hurdle for many patients in India. The average annual expenses spent on Diabetes related healthcare costs is around Rs. 30 thousand, which translates to Rs. 2500 per month [23]. Considering the price sensitive Indian diabetes market, this amount is beyond reach for many diabetics and thus warrants the use and acceptance of more economical biosimilar versions of available GLP-1RA medications. Liraglutide biosimilar is priced at 1/3rd the cost of reference liraglutide, addressing the core issue of financial implications associated with the use of GLP-1RA therapy. Therefore, Liraglutide biosimilar is both an economical and effective treatment modality for Indian Diabetic patients suffering from overweight and obesity, with definitive weight loss benefits.

## Conclusion

Biosimilar Liraglutide at a dose of 1.8 mg resulted in significant weight reduction and glycemic control (HbA1c, FPG and PPG) from baseline to end of study in T2DM patients with obesity. The weight reduction was comparable to reference liraglutide at the end of study.

## Electronic Supplementary Material

Below is the link to the electronic supplementary material.

Supplementary Material 1

## Data Availability

No datasets were generated or analysed during the current study.
