# Correction: Effect of liraglutide biosimilar vs. reference liraglutide on weight reduction in T2DM patients with obesity: post hoc analysis of phase III trial

**DOI:** 10.1186/s40842-025-00233-9

**Published:** 2025-08-19

**Authors:** Sujoy Ghosh, Bipin Sethi, Sanjay Kalra, Manash P. Baruah, Abhishek Mane, Sanjay Choudhari, Anup Petare, Mayur Jadhav, Saiprasad Patil, Hanmant Barkate

**Affiliations:** 1 Manipal Hospitals, Kolkata, India; 2 Care Hospitals, Hyderabad, India; 3 Bharti Research Institute of Diabetes & Endocrinology, Karnal, India; 4 Apollo Hospitals, Guwahati, India; 5 Glenmark Pharmaceuticals Ltd, Mumbai, India

**Correction**: *Cardiovasc. Diabetol. – Endocrinol. Rep.* **11**, 6 (2025). 10.1186/s40842-025-00219-7

Following publication of the original article it was reported that there was an error in the ‘Competing Interests’ declaration and the ‘Funding’ declaration. The previous and updated declarations are given below.

Original ‘Funding’ declaration: We thank both Levim Biotech LLP & BIRAC (Biotech Industry Research Assistance Council) of DBT, GOI for their financial assistance with respect to the conduct of Liraglutide Biosimilar Phase III clinical trial.

Updated ‘Funding’ declaration: We acknowledge Levim Biotech LLP, Biotechnology Industry Research Assistance Council (BIRAC), Department of Biotechnology (DBT), India for their financial assistance towards the conduct of the Liraglutide Biosimilar Phase III clinical trial.

Original ‘Competing interests’ declaration: The authors declare no competing interests.

Updated ‘Competing interests’ declaration: Abhishek Mane, Sanjay Choudhari, Anup Petare, Mayur Jadhav, Saiprasad Patil and Hanmant Barkate are employees of Glenmark Pharmaceuticals Ltd. While Glenmark was involved in aspects of manuscript support and data interpretation, all efforts were made to ensure the scientific integrity and objectivity of the study findings.

Furthermore, the following Acknowledgement declaration has been added:

**Acknowledgements**

We thank the Glenmark Pharmaceuticals team members for their support in manuscript writing and review.

