# Supplementary figures and images for: Effect of liraglutide biosimilar vs. reference liraglutide on weight reduction in T2DM patients with obesity: post hoc analysis of phase III trial
Source: Cardiovasc Diabetol Endocrinol Rep. 2025 Mar 25;11:6. doi: 10.1186/s40842-025-00219-7 (PMC11964147; doi:10.1186/s40842-025-00219-7)

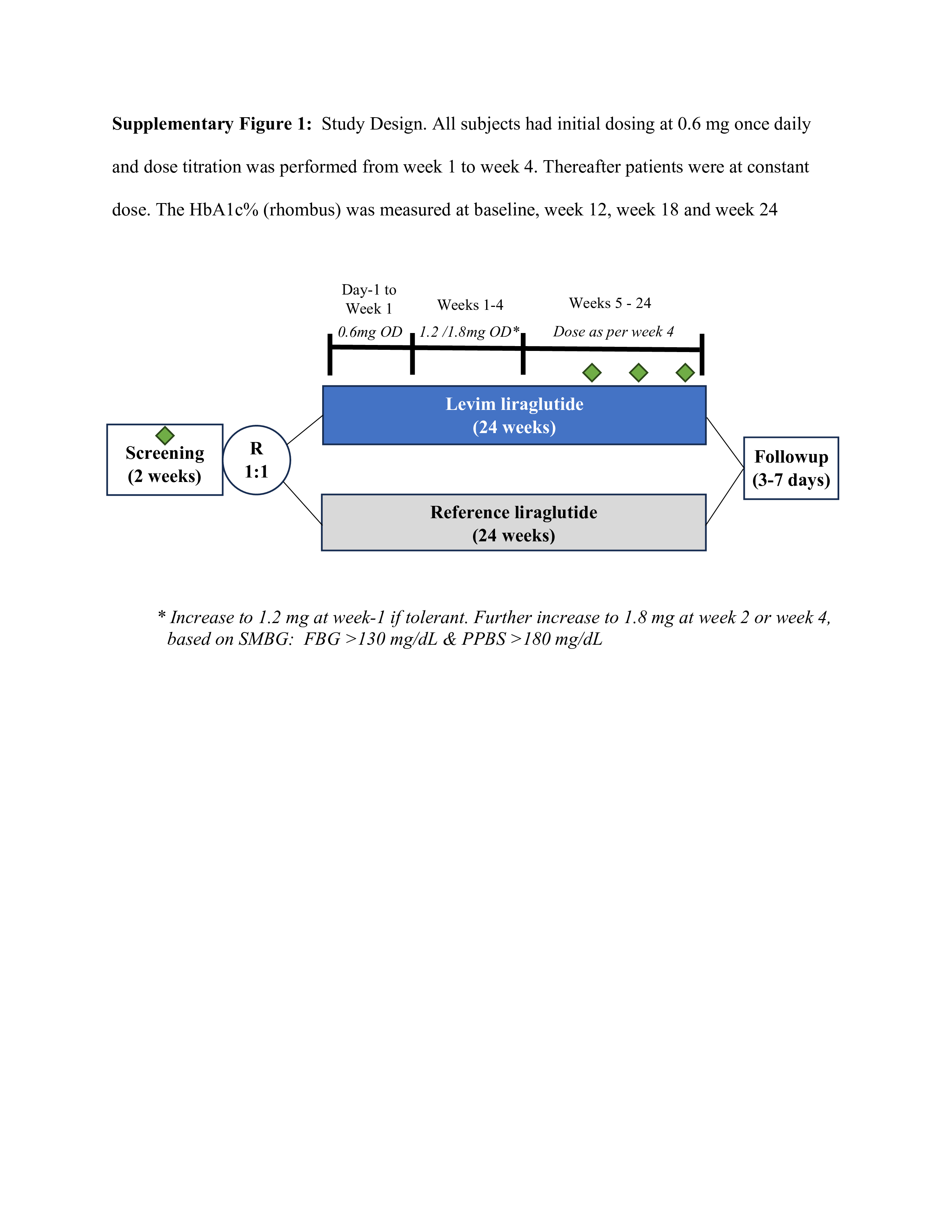

Supplement: Supplementary file 1 — Supplementary Material 1 [file 40842_2025_219_MOESM1_ESM.jpg]
